# Crystallography in Open Science and its open educational resources

**DOI:** 10.1107/S2053273326004146

**Published:** 2026-06-16

**Authors:** John R. Helliwell

**Affiliations:** a Department of Chemistry University of Manchester Manchester United Kingdom; Institute of Crystallography - CNR, Bari, Italy

**Keywords:** foundations of crystallography, Open Science, Open Data, open educational resources, central facilities, databases, data archives, science policy, global perspectives, citizen science

## Abstract

This article presents a review of the landscape of Open Science development and its relevance to crystallography. As open as possible as closed as necessary is the model for Open Science as commended by the UK and China. Mainland Europe requires compulsory release after 3 years since measurement of raw data. The USA policy situation has fluctuated considerably in recent years. Altogether, this article addresses the International Union of Crystallography’s (IUCr) input into these activities. It considers its participation in international scientific and societal organizations, as well as its compliance with the policy recommendations. It considers the practical landscape in which crystallographers work, including their funding agencies, governments, central facilities and universities. This article sets the wider scene before describing how crystallography benchmarks against those under development. The IUCr’s Teaching Pamphlets and the Online Dictionary of Crystallography are educational resources fully open to Global South and Global North readers and authors. Crystallography provides a mature, practice-based model of responsible openness that predates and can inform contemporary Open Science policy.

## Abstract 2

Open Science is the movement to make scientific research, the data and their dissemination available to any member of an inquiring society, from professionals to citizens, irrespective of their economic situation. The IUCr provides fully open educational resources to Global South and Global North readers and authors.

## 1. Introduction

Open Science fosters the development of open infrastructures and tools such as open-source software and open data repositories. It has, in many ways, paved the way for today’s advancements in artificial intelligence (AI) and computing by making software and data accessible and providing a foundation for others to build upon. The FAIR (Findable, Accessible, Interoperable and Reusable) principles were introduced by Wilkinson *et al.* (2016 ▸), who also coined the acronym. However, the underlying practices embodied by FAIR have a much longer tradition within crystallography. W. L. Bragg in his first paper on crystal structures (Bragg, 1913 ▸) showed the raw diffraction data, both the Laue X-ray diffraction photographs and the monochromatic X-ray rocking curves from the alkali halide single crystals. The Cambridge Crystallographic Data Centre from its inception in 1965 pioneered FAIR data with careful metadata; its eventual transition to a subscription-funded, trusted archive shows the difference between FAIR and Open Data. See https://www.ccdc.cam.ac.uk/discover/blog/coretrustseal-certification-the-csd-as-a-trustworthy-data-repository/. In a complementary approach, the Crystallography Open Database enables unrestricted download of its full collection for reuse and analysis (Gražulis *et al.*, 2009 ▸).

Open Science is therefore deeply connected with crystallographic practice. For example, *AlphaFold*, which made groundbreaking contributions to predicting protein folds from amino acid sequencing, was made possible, in part, by the Protein Data Bank (PDB) and the broader genomic revolution it helped initiate, as well as clever algorithms developed by the AI community in general and Google *DeepMind* in particular (Jumper *et al.*, 2021 ▸). It was the PDB that laid the groundwork for fully open data sharing by crystallographers with its infrastructure, careful metadata capture and collaborative research practices in genetics and molecular biology (Burley *et al.*, 2017 ▸). 90% of the macromolecular crystal structures in the PDB, that underpin *AlphaFold*, are from synchrotron radiation (SR), the culmination of decades of effort from multiple SR facilities in the world (Helliwell, 2026 ▸). Open data are widely recognized as a prerequisite for AI/ML (machine learning) to proceed (Lawrence & Montgomery, 2024 ▸). In general, repositories must earn the trust of the communities they intend to serve and demonstrate that they are reliable and capable of appropriately managing the data they hold. Lin *et al.* (2020 ▸) describe the TRUST principles (Transparency, Responsibility, User focus, Sustainability, Technology).

Arguably, the most visible success of openness is *Wikipedia*, now operating for 25 years. As noted in a recent *Nature* editorial (Skipper, 2026 ▸), it is free to use, highly participatory and grounded in citation transparency across its roughly 65 million entries. Its co-founder Jimmy Wales described it as ‘a worship of expertise’ (Wales, 2026 ▸; Wales, 2025 ▸). While entries require critical evaluation, *Wikipedia* demonstrates that large-scale open knowledge production can function sustainably and at global reach.

## 2. Definitions

### 2.1. A varying landscape

UNESCO and the International Science Council define Open Science as (International Science Council, 2025 ▸):

Open Science is the movement to make scientific research, data and their dissemination available to any member of an inquiring society, from professionals to citizens (ORION Open Science, 2017 ▸). Open scientific knowledge (of which data are a part) is a cornerstone of the 2021 UNESCO Recommendation on Open Science (UNESCO, 2021 ▸). The adoption of Open Science practices has helped accelerate scientific discovery. Examples include the Human Genome Project, which publicly released genomic data and led to major breakthroughs in medicine and genetics, the Sloan Digital Sky Survey, which made high-quality astronomical data freely available and transformed research in astrophysics, and the Copernicus Data Space ecosystem, which provides access to geospatial data from the Copernicus Sentinel missions. Open Science promotes a broad cultural shift and encourages reproducibility, collaboration, access to and inclusion in scientific research. The FAIR approach to data is part of the Open Science landscape and can help improve scientific research practices. These approaches strive to make data more transparent, accessible and usable for the scientists and the general public (Umbach, 2024 ▸).

A difficulty of the above definition is that the examples quoted involve studying objects of nature, namely the human genome and the night sky. Crystallography involves crystallization and other sample conditions devised by a person and who is therefore *de facto* the intellectual property owner. Of course this connects with the pharmaceutical industry. In most legal jurisdictions a naturally occurring molecule found in nature (without modification) cannot be patented. Molecules are patentable if novel and non-obvious. Polymorphs are sought as they can vary in their solubility and thereby may be of benefit to a patient. A unique powder X-ray diffraction pattern is the necessary evidence for a novel polymorph. To come back to the aspect of definitions ‘open by default’ could undermine patent filing windows, whereas ‘as open as possible as closed as necessary’ protects innovation while enabling later openness. In pharmaceutical crystallography, timing of disclosure is legally critical.

It will already be obvious that there is more than one presumption of what open science means. But, also, the published literature acknowledges this: Fecher & Friesike (2014 ▸) analyse five schools of thought, ‘Open Science is an umbrella term encompassing a multitude of assumptions about the future of knowledge creation and dissemination’.

This observation remains relevant today. Discussions at the UN Open Science and Open Scholarship conference in Tokyo in October 2025 openly acknowledged the diversity of interpretations and expectations surrounding Open Science (*e.g* Chuan-Peng, 2025 ▸). At that meeting, the concept of ‘responsible Open Science’ also emerged strongly, emphasizing that openness needs to be considered within differing scientific, social and ethical contexts.

This contextual perspective is consistent with the UNESCO (2021 ▸) Recommendation on Open Science, which states that scientific knowledge should be ‘as open as possible and as closed as necessary’. The recommendation further recognizes a range of justified restrictions – including privacy, intellectual property, human subjects, Indigenous knowledge and security considerations – and explicitly notes that such conditions may evolve over time. While some Open Science advocacy communities and conference discussions may strongly promote ‘open by default’, UNESCO’s formal (2021 ▸) policy framework is broader and more contextual in nature.

I need to offer some notes on a few nuances of definition. Firstly, where open science is a reference to a recommendation or the movement, I capitalize it, *i.e.* as Open Science; otherwise, where it can have multiple meanings, as I described above, I refer to open science in lower-case letters. Secondly, I apply a similar consideration to Citizen Science, where usually it means the Citizen Science movement; likewise Open Data. However, where historical precedent stretches back many decades lower case is usually used. Thirdly, the definitions of Global South and Global North are economic descriptors of the geopolitical relations of power, revealing the root cause of said economic differences. The simplest practical pr­oxy often used is that OECD members are approximately the Global North comprising 38 countries as of 2025 and the non-OECD countries form the Global South. But this is imperfect as OECD membership also includes Mexico, Chile, Colombia, Costa Rica and Türkiye, which are not always classified politically as Global North. So, OECD membership is not strictly identical to Global North. A more detailed description of their use and inadequacies as terms today can be found in Sims (2024 ▸).

### 2.2. Sharing of data

Open data sharing, as embodied by the FAIR principles (Wilkinson *et al.*, 2016 ▸), is a key component of the Open Science movement. FAIR does not mean open but is a necessary, albeit insufficient, need. Open data involves no paywall, allowing data to be easily accessed and reused, facilitating research reproducibility and efficiency. However, making open data accessible relies on physical storage infrastructures and a skilled workforce to curate the data, apply metadata and manage the associated semantic resources. Strengthening these skills within the scientific community will help to realize the gains described above. Open science provides a framework for data to be harnessed for AI for science, but care should be taken so that open data are not employed for perverse or nefarious purposes.

There are also many types of data that are usually quite well defined. Scientific data are data generated by experiments and observations as part of the scientific process. These encompass raw data, which become the processed data, ultimately giving rise to a derived model. We assume that data are in a digitized, machine-readable format.

### 2.3. A very important special case, the Indigenous Peoples

The CARE principles (Collective Benefit, Authority to Control, Responsibility, Ethics) (Research Data Alliance, 2019 ▸) were developed to assert Indigenous data sovereignty and the need for data gathering and access to be bounded by ethical considerations and respect. CARE highlights that data collected from or about Indigenous Peoples should also be governed and shared on Indigenous Peoples’ terms (Carroll *et al.*, 2022 ▸). The ethical principles of CARE have wide applicability. The FAIR principles are explicitly focused on technical issues: how to describe data and metadata to make them machine actionable. This includes specifying the conditions under which protected data might be accessed. CARE provides a complementary framework to FAIR towards responsible data sharing (https://ukdataservice.ac.uk/learning-hub/research-data-management/plan-to-share/care-data-principles/). If applied in conjunction, they can support the principles of Open Science and help mitigate safety risks. Overall, the United Nations Declaration on the Rights of Indigenous Peoples (UNDRIP) is a landmark non-binding resolution adopted by the UN General Assembly on 13 September 2007 (https://www.un.org/development/desa/indigenouspeoples/wp-content/uploads/sites/19/2018/11/UNDRIP_E_web.pdf). It affirms the collective and individual rights of Indigenous Peoples worldwide, establishing minimum standards for their survival, dignity and wellbeing.

### 2.4. Data quality

Is data quality defined? This varies by field which is said to be why FAIR does not state anything about data quality, but it does require adequate metadata for reusability and interoperability. High-quality data sets are accurate, complete, verifiable and contain the data needed to answer a specific question or set of questions (National Center for Advancing Translational Sciences. See *e.g.* the guide on data quality at https://toolkit.ncats.nih.gov/glossary/data-quality/). See also Hackert *et al.* (2016 ▸).

Verhulst *et al.* (2025 ▸) address the readiness of data for successful AI:

Efforts (are needed) for enhancing AI-ready data through improved data labelling, provenance tracking, and new data standards. However, key challenges remain: How can data be structured for AI without compromising ethics? What governance models ensure equitable access? How can AI itself be leveraged to improve data quality? Answering these questions is essential for unlocking the full potential of AI-driven innovation while ensuring responsible and transparent data use.

Work is ongoing in this field and in responsible AI. The different kinds of licences for data are described at https://data.europa.eu/en/news-events/news/understanding-open-data-licensing-insights-18-million-datasets.

### 2.5. The modern target to reach is set as Diamond Open Access

An increasingly popular term in publishing is Diamond Open Access: free for authors and free for readers. For a publication to be defined formally as Diamond Open Access, all its outputs need to carry an open licence, such as a Creative Commons licence (https://zenodo.org/records/15128179, https://creativecommons.org/share-your-work/cclicenses/). Also, it is important to note that ‘open data’ is not a free-for-all, and that reusing open data requires proper attribution to the original experimenters, in line with relevant licence terms.

## 3. The global Open Science landscape

### 3.1. UNESCO and international policy developments

UNESCO’s efforts in this area appear primarily aimed at understanding and addressing a world divided by economic resources. UNESCO seeks to find ways to reset these obvious asymmetries across the globe. Within this, UNESCO is very cautious about commercialization which leads to power asymmetries between the Global North and the Global South. This term captures the important point that consumers are split into the two geographic regions of the World, one where they can afford to pay for products or medicines and another where they cannot afford to. One of the most powerful examples of power asymmetries shown at the recent United Nations 4th Conference on Open Science and Open Scholarship Conference held in Tokyo in October 2025 was the world mapped according to publication citations as shown by Leslie Chan of the University of Toronto (Chan *et al.*, 2011 ▸). This highlighted the striking imbalance in citation patterns, with publications from the Global North rarely referencing work originating elsewhere. Furthermore, it suggests that science does not achieve a global public good, ‘only’ a Global North public good. It perhaps gives insights into the approach taken by UNESCO and the International Science Council in seeming to promote ‘open by default’ at its events through its choice of speakers.

The basics of public good theory are long-established. Chin (2021 ▸) of the International Monetary Fund summarizes this theory and concisely articulates that global institutions must coordinate to preserve the goods that benefit us all.

In UNESCO and UN discussions I frequently encountered the term ‘private actors’, though its meaning was often ambiguous. It may refer to industry, investors, non-governmental organizations, or even citizen scientists. The term appears to carry different connotations across institutions. UNESCO tends to emphasize ethical guidance in view of global power asymmetries; the UN frames private actors as development partners; the World Bank views them as investors and service providers; and the OECD treats them as regulated market participants within member economies. These differences help explain variations in Open Science policy emphasis.

There remains the question of monitoring the uptake of Open Science recommendations. UNESCO have taken the initiative on this. There are several organizations involved which have so far opted to ‘only’ monitor the take-up of the ‘principles of Open Science’ in their OSMI (Open Science Monitoring Initiative, https://open-science-monitoring.org/). In the UNESCO July 2025 conference on Open Science (https://open-science-monitoring.org/7-and-8th-july-2025-open-science-monitoring-progress-assessing-impact/) there was a repeated view from the audience, with which I agree, that actual metrics are needed.

### 3.2. Open Science and the UN Sustainable Development Goals

#### 3.2.1. Neglected tropical diseases

In the broadest sense, open science would address neglected areas like tropical disease drug development, which lacks traditional market incentives for industry. In such cases, a fully open collaborative approach [*e.g.* as powerfully argued by Miedema (2022 ▸)] could accelerate breakthroughs. Another situation can involve pre-competitive, open, collaborations between companies, such as the Industrial Macromolecular Crystallography Association’s beamlines at the Advanced Photon Source (https://www.imca-cat.org/About). Also, for very urgent disease challenges to society such as Covid-19, industry and academia can work fully openly and/or not-for-profit. A hybrid approach such as the Covid Moonshot contributed to the rapid development of Ensitrelvir by the company Shionogi, underscoring how transparent scientific exchange can directly speed up therapeutic innovation (Boby *et al.*, 2023 ▸). Preprints have been a focus for the funding agencies in recent years but risk undermining trust and even misleading as the associated data are rarely provided with a preprint (Helliwell, 2024 ▸) (see below).

#### 3.2.2. Openness and innovation in industry

Efforts to encourage innovation in open ways are being actively explored. Svendsen *et al.* (2025 ▸) observe that

Europe’s investment in cutting-edge Research Infrastructures (RIs) is critical to maintaining scientific leadership and tackling major societal challenges. However, these investments do not automatically translate into innovation. Strengthening collaboration between RIs and industry is essential to unlocking the full value of RIs, but persistent barriers – including lengthy Intellectual Property (IP) negotiations, bureaucratic hurdles, and cultural divides – often hinder effective engagement.

Furthermore that

Even though open access is often a possibility (and is required in EU-funded projects), most other collaborations use closed modes by default.

The uptake of open science resources in industry may well vary *e.g.* according to the size of a company, from the small and medium enterprises (SMEs) through to large conglomerates. The EU recognizes that more information is needed on how industry accesses these resources (https://on-merrit.eu/news/2021-12-16-os-resources-industry/#fnref:2).

**Figure 1:**
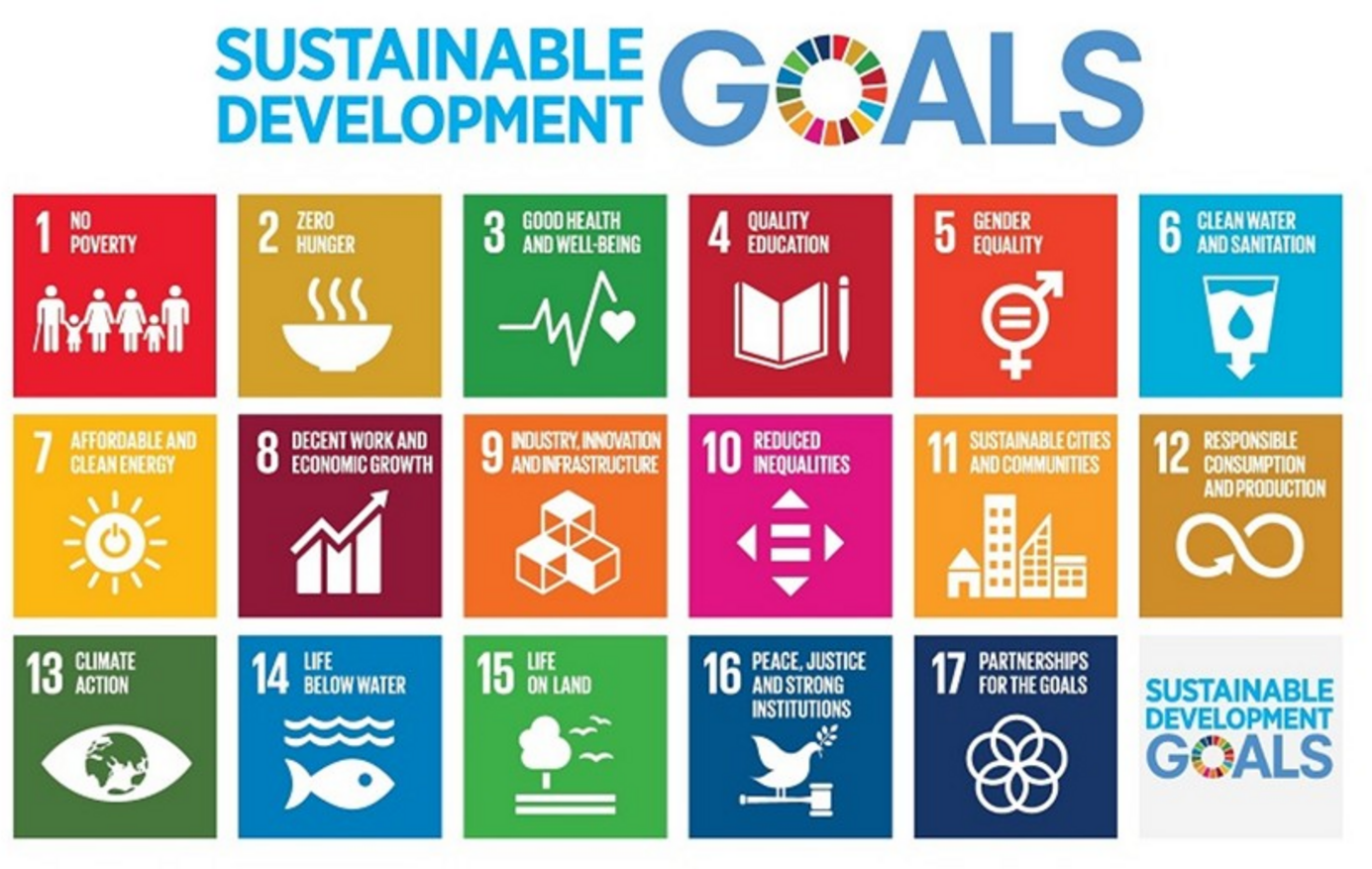
The UN’s Sustainability Development Goals (United Nations, 2015 ▸).

UNESCO’s Recommendation on Open Science (https://www.unesco.org/en/open-science/about?hub=686) often underrepresents industry’s role, despite its prominence in addressing societal challenges, as emphasized by the UN’s SDG9 (where SDG stands for sustainable development goal) (https://www.globalgoals.org/goals/9-industry-innovation-and-infrastructure/) and SDG8. The UN’s SDGs aim to achieve a marked change in the world for the better (see Figs. 1 ▸ and 2 ▸).

#### 3.2.3. Industry and synchrotron beamlines support

To close this section, I remind readers that industry leverages facilities like synchrotron services under strict confidentiality, which is essential to their innovation process. Supporting industrial access to synchrotron facilities was an important component of operations at the SRS Daresbury laboratory during its operational life between 1981 and 2008. Likewise, I have commended this in my advisory roles at all the newer synchrotron, and neutron, facilities, encouraging them to provide access to industry. More broadly, industry plays an essential role as a contributor to societal well-being and innovation. Consumers of course need to be critical of the products on offer and governments need to regulate to protect the consumer including stopping monopolies – otherwise competition between products would cease.

### 3.3. Governmental and funding agency approaches

This is a big topic to summarize. The discussion here is primarily informed by experience within the UK research system. But, in general, I expect all governments to take up the two distinct roles of both regulation setting and economic development; a major review of the UK universities and business interface is the Lambert Review in 2003 (https://dera.ioe.ac.uk/id/eprint/16532/1/Lambert%20Review%20of%20Business-University%20Collaboration.pdf). In simple terms, open science is likely to be a requirement for the small and medium enterprises, vital for enhanced growth and the country’s productivity but less so for the large corporations with substantial finances and internal research budgets.

The UK, EU and US approaches differ overall. My observations suggest that the US is very concerned with respecting the intellectual property rights of individuals. At the other extreme, the EU sees publicly funded research as meaning it has a power on enforcing compulsory raw data release as a duty to taxpayers. Their basic motivation is that publicly funded research belongs to the public and should not be behind a paywall. This makes sense if one believes that the public is paying for the research and understands the research involved. The counter-argument is that the public places its trust in its principal investigators, provided their proposals are approved by the funding agencies as their proxies. Different parts of the world adopt one of these two positions. Some place primary trust in principal investigators, others regard the taxpayer as having ultimate ownership and responsibility. Climate change research illustrates this tension. Public trust may be placed either in the scientific community or in political leadership when interpretations differ. This highlights the broader question of where authority over scientific evidence ultimately resides.

The UK adopts a hybrid approach, most neatly summed up in the UKRI’s data policy (https://www.ukri.org/manage-your-award/publishing-your-research-findings/making-your-research-data-open/), to respect intellectual property as a closed where necessary category. The ISIS neutron facility data policy (https://www.isis.stfc.ac.uk/using-isis/academics/policies/) is an example that contradicts the statement that all UK facilities follow the ‘the PI decides if data are open’ principle (where PI is principal investigator).

The EU’s approach is described in its document European Commission: Directorate-General for Research and Innovation, *Open innovation, Open science, Open to the world – a vision for Europe*, 2015, https://data.europa.eu/doi/10.2777/061652. The USA White House announcement on Open Science under the Biden administration is available at https://bidenwhitehouse.archives.gov/ostp/news-updates/2024/01/31/fact-sheet-biden-harris-administration-marks-the-anniversary-of-ostps-year-of-open-science/. The White House is changing its policy, the new one called Gold Standard Science also calls for making evidence public but puts constraints on what is considered Gold Standard Science (https://www.whitehouse.gov/presidential-actions/2025/05/restoring-gold-standard-science/).

**Figure 3:**
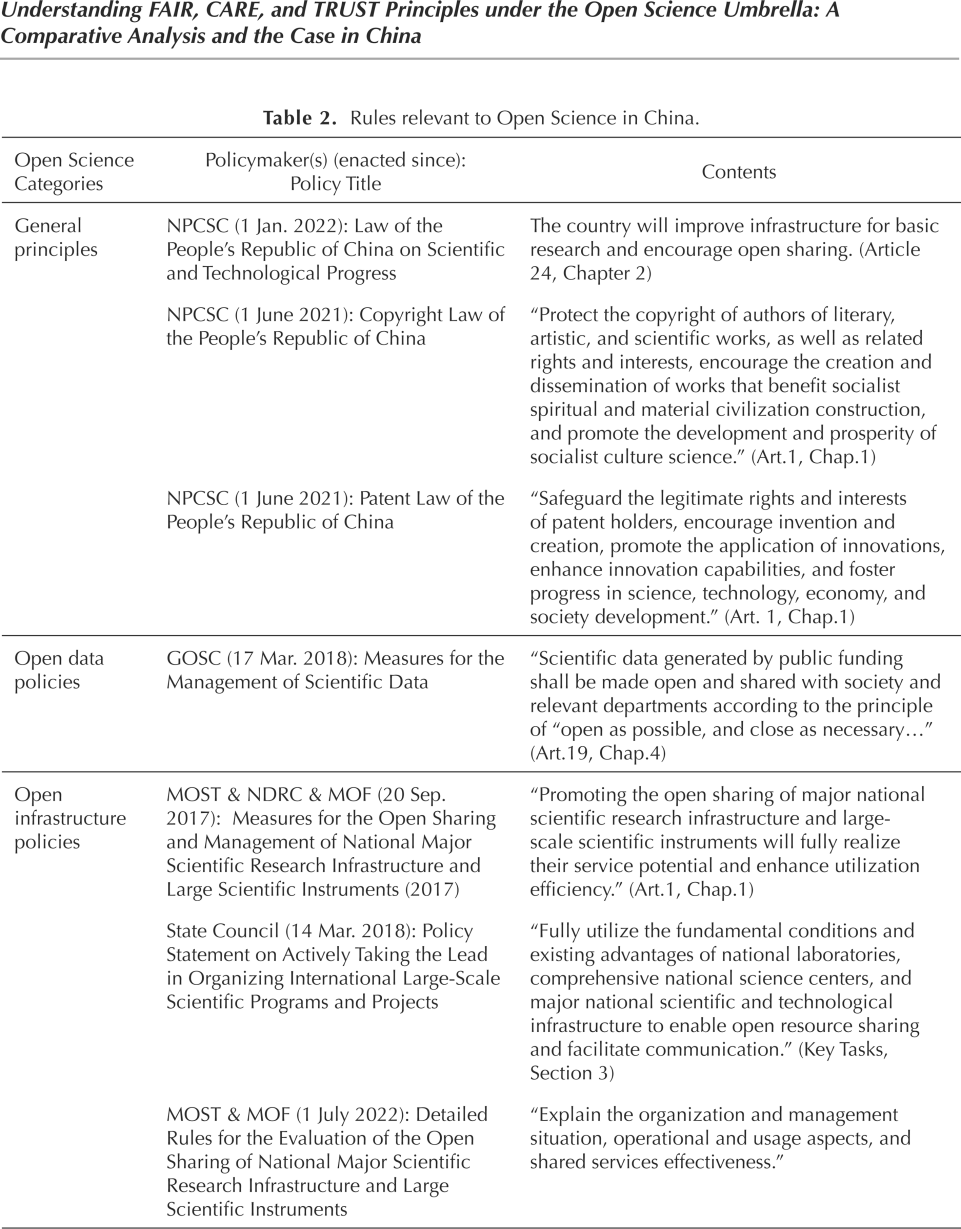
This illustrates China’s very careful approach to protect copyright and patents whilst signalling their adherence to ‘as open as possible as closed as necessary’. From Zhang (2025 ▸) with permission.

In the above, I have compared UK, EU and the US. What do we know about China’s approach to Open Science? This is described in detail by Zhang (2025 ▸) within which is the summary in Fig. 3 ▸ (Table 2 in Zhang’s article).

### 3.4. Data ownership, PI responsibility and the spectrum of openness

#### 3.4.1. Open by default examples

This topic relates to the general policy options of ‘Open by default’ or ‘As open as possible as closed as necessary’ and within them the PI responsibilities. More bluntly, we can pose the question: who owns, *i.e.* controls, the data?

There are clearly cases where every effort should be made to insist on open science. The most obvious is for publications on new pharmaceuticals and their clinical trials. Clinical trials are a special case, where ethical obligations to participants and the public justify stronger openness requirements than in many areas of industrial R&D. Within this, patient/participant confidentiality is paramount and requires many levels of security to prevent the release of personal data. The *British Medical Journal*, *BMJ*, is leading the way by insisting in their Notes for Authors (Loder *et al.*, 2024 ▸):

We hope that you will support us in enhancing transparency and scrutiny of medical research. It is important to put the public good ahead of personal, academic, and corporate interests.

Unimpeded access to both data and code across the research community maximizes the value of each research project. It shows respect for the efforts of research participants and the economic contributions of the public.

The *BMJ* represents the most ambitious end of the clinical trials transparency spectrum, while the *New England Journal of Medicine*, *The Lancet* and regulators emphasize controlled access and proportionality – reflecting the practical realities of clinical research where corporate entities protect their large financial investments. Where the *BMJ* quotation above raises a particular concern is that it could violate patient confidentially when it states ‘*put the public good ahead of personal…interests*’.

An interesting example of rights ownership is where a group of PIs in a field decide, before anything is measured, that their measurements will be made open. The public Human Genome Project decided on this as mentioned above. Additionally, the European Synchrotron Radiation Facility (ESRF) has such an example with its human organ atlas (HOA) imaging project (https://human-organ-atlas.esrf.eu/). Its data are made available under the Creative Commons Attribution CC BY 4.0 licence. This means the data sets are accessible by all for reuse as long as the DOI (digital object identifier) is cited when the data are used. Then, any publications using HOA data must cite the data set DOI from every data set used in the publication. The DOIs can be found on the data set’s web pages. The ESRF also encourages authors to cite other relevant papers, such as the paper describing the experimental methods used to produce these data sets (Walsh *et al.*, 2021 ▸).

#### 3.4.2. Open by default after 3 years examples

A key feature of mainland Europe’s central facilities data policy is that after an embargo period of 3 years all measured data are made publicly available with one DOI per experiment. Applicants for beamtime sign up to this policy when they apply. There is scope for appeal to extend the embargo period (see below for what this is, which is at the discretion of the facility).

It is an important development that central facilities like the ESRF have moved to being extremely bright sources (EBS) due to their improved accelerator machine lattices. Modern EBS allow the study of ever weaker scattering objects, approaching single molecules rather than macroscopic crystals. However, they also generate enormous data volumes, overloading PIs who may be unable to analyse all results fully. This is especially true at these EBS where the data rate continues to increase and hundreds to tens of thousands of macroscopic samples can be measured in some cases. As noted by Andy Goetz at the ESRF:

Taking just ESRF as an example we have recorded raw data for 17365 experimental sessions producing 6450651 datasets over the last 5 years, *i.e.* more than 90% of the data in our repository…. PIs are overloaded with data and do not have enough time to analyse all their results or do justice to all the data. This is especially true at synchrotrons where the data rate keeps on increasing and hundreds to thousands of samples can be measured in some cases. Making the data open after the embargo period has two major positive effects which are not related to mistrust of the PI: (1) the facility takes care to provide better metadata because they are being preserved for posterity, and (2) PIs take more care to use the data because they know there is an embargo period. They can also extend the embargo period if they need to. At the ESRF we have had one request for this.

This approach is governed by mainland Europe’s Photon and Neutron Open Science Cloud (PaNOSC) policy (Murphy *et al.*, 2025 ▸). A summary is available (Goetz, 2020 ▸) where 22 main points of the new policy are listed, of which five concern the embargo period concept. It is a major shift in facilities’ policies on data that mainland Europe is taking. Key to this is the concept of automatic release by the facility of the raw data appropriately registered with a DOI. The facility is, in effect, challenging the PI’s decision as to when a study is mature enough to be reported. The PaNOSC data policy likewise recognizes that it is responsible to taxpayers to make available all data irrespective of its maturity. There is a conflict of approach here. From mainland Europe’s view there are advantages, listed above, in automatic release of raw data where there is no publication at the 3 years’ point. The duties of a PI are summarized in the supporting information, as seen through my eyes as a crystallographer PI since 1979. I have also served as synchrotron facility director of the UK’s SRS, and I can see explicitly the concern of the central facilities at the fraction of unused raw diffraction data. This concern also needs to be recognized by our community. One approach would be for the facility to harness the expertise of the PI and have them score their raw data sets to assist the training of the AI and ML envisaged to trawl through a facility’s raw data archive.

#### 3.4.3. Pre-registration of studies is a growing theme

Pre-registration is the practice of registering the hypotheses, methods or analyses of a scientific study before it is conducted. In the standard pre-registration format, researchers prepare a research protocol document prior to conducting their research. In the clinical sciences I am surprised to see that pre-registration is only slowly being adopted, given that negative clinical trials and their raw data obviously need to be out in the open (Nilsonne *et al.*, 2025 ▸). The persistence of such problems in medical research highlights the complexity of ensuring transparency and accountability across different scientific domains. Of course, some medical discoveries do not lend themselves to pre-registration such as the accidental discovery of penicillin by Sir Alexander Fleming (1929 ▸). An extensive description of this growing practice in science as a whole is described at https://en.wikipedia.org/wiki/Preregistration_(science).

### 3.5. Historical case study of data ownership and PI responsibility: data ethics and Photo 51

An example of bad judgement on open science is the controversial use by James D. Watson and Francis Crick of the X-ray diffraction photograph of a particular hydrated state of DNA taken by Franklin & Gosling (1953 ▸). [‘Photo 51 was taken by Raymond Gosling (a PhD student) working under Rosalind Franklin’s supervision, on 2 May 1952’ (Wikipedia)]. Under today’s rules governing reuse of raw data, even the most liberal policies provide a defined period of confidential use. (For example, see https://www.esrf.fr/cms/live/live/fr/sites/www/home/UsersAndScience/UserGuide/esrf-data-policy.html.) Only after that period are the data released, unless an appeal is granted by the facility director. From the perspective of contemporary data-use norms, the use of Photo 51 by Watson & Crick (1953 ▸) appears to be controversial. Questions about the ethics of data use in this episode continue to be debated, affecting historical perceptions of both Watson and Crick.

The Watson & Crick (1953 ▸) paper describing their model of the double helix of DNA was momentous because, as they themselves put it, ‘It has not escaped our notice that the specific pairing we have postulated immediately suggests a possible copying mechanism for the genetic material’. Just how momentous? I would say it was the most significant scientific discovery of the 20th century.

I would label the DNA diffraction photograph sharing as a bad judgement case of forcing open science by default, disregarding the intellectual property (IP) of Ray Gosling and Rosalind Franklin.

### 3.6. Preprints and peer review in an open era

Preprints are promoted actively by the International Science Council (Drury, 2022 ▸). The pioneer of preprints, from the physics community, was *arXiv* (https://arxiv.org/).

A past president of the International Union of Crystallography (IUCr), Professor Philip Coppens, consulted me as to whether the IUCr should introduce preprints for crystallography. Ultimately, the absence of underlying data and peer review in preprints led to the view that introducing crystallography-specific preprints would be problematic. In discussing this problem with PDB pioneer Phil Bourne at the International Data Week in Denver in 2016, he swept my objection aside, stating flatly that a preprint with no attached data was better than no preprint at all; see McMahon & Helliwell (2026 ▸).

### 3.7. Citizen Science and public participation

Citizen Science projects are keen on all measured data being made open, even from the moment of measurement, like the Human Genome Project did. This would include research on neglected tropical diseases, which relies principally on academic efforts rather than industry. Citizen Science as an approach is especially harnessed in astronomy night sky surveys and ecological surveys of, for example, numbers of plastic containers found on beaches. There are and likely to be many other examples (Cole *et al.*, 2024 ▸). A challenge here, with very large numbers of contributors of data, is how to understand and ensure data provenance and integrity so as to get the greatest value from making these data open.

### 3.8. Reproducibility, replicability and data quality

The international academies have played a key role in defining reproducible and replicable science and extending this to the case of disaster risk management. More widely in science it seems common that the terms reproducibility and replicability are used interchangeably, when they are distinctly different. These terms are very well described by the US National Academies report (2019 ▸). Replicability means that a study can build on previous studies’ findings to see if it can replicate the previous ones. Detailed checks of the original study, usually its underpinning data and calculations, are needed first to ensure its findings were reproducible.

In the *History of Scientific Journals* (Fyfe *et al.*, 2022 ▸) there is an account of an interesting contrast between the Paris Académie Royale’s editorial committee overseeing its Histoire et Mémoires in the 18th century and the Royal Society’s publications:

In Paris, at least by the later eighteenth century, the academicians scrutinized the observations submitted to them by outsiders in great detail. This process could take a year or more and could involve checking the experiments or asking for additional experiments, as well as requesting (or making) revisions to the paper. In London, the Royal Society’s committee operated quite differently. First, the focus at the committee meetings was not usually the full text of the paper, but the ‘minute’ of each paper recorded by the secretary in the Society’s ‘Journal book’ as a record of the Society’s meetings. These summaries were read to the committee, in order, ahead of each vote. Any committee member could request that the entire paper be read out, but the norm was theoretically a vote based on the secretary’s summary plus any personal recollection of hearing the paper at an ordinary Society meeting sometime in the previous month. (Thus,) papers could appear in the (Royal Society’s) Transactions within six months of submission, whereas mid-eighteenth century volumes of Histoire et Mémoires generally appeared two or three years after the year actually printed on the title page.

The Paris Académie Royale’s editorial committee approach therefore added replicability checks before agreeing publication since in that pre-digital age reproducing a study via the underpinning data and the reanalyses of those data was obviously not viable as it is today.

A much-mentioned area for the benefits of open science is that of best management of disasters and specifically cited is that of the challenges of Covid-19. Macromolecular crystallography made a substantial and very positive contribution here both in facilitating finding new drugs (see *e.g.* Boby *et al.*, 2023 ▸) and more immediately in guiding the development and improvement of vaccines. There was a tension apparent between rapid delivery of results by the community and the need for their close examination and in many cases corrections. An overview of the Covid-19-related proteins examined by the Coronavirus Structural Task Force was described, after the Covid-19 crisis, in a wide range of topical reviews that they published in *Crystallography Reviews*. An editorial overview has been provided by Cianci *et al.* (2026 ▸). The wwPDB coordinates versioning protocol introduced a few years ago enabled effective improvement of structures archived in the PDB. As of January 2026, there were 975 entries with coordinate replacements for various reasons and, overall, the PDB archive housed a total of 5588 SARS-CoV-2 structures. The top reasons for updating were model completeness, ligand identity, model orientation/position and ligand geometry (Jasmine Young, RCSB, personal communication).

In general, as introduced earlier in this article, the issue of data quality has not been described in the FAIR data accord (Wilkinson *et al.*, 2016 ▸). This led to pressure from the numerous representatives to CODATA (Committee on Data of the International Science Council), including myself as the IUCr Representative between 2012 and 2023, to recognize the importance of data quality.

CODATA has recently (2025 ▸) launched a Task Force on Data Quality (https://codata.org/initiatives/task-groups/research-data-quality-management-across-the-data-lifecycle/). CODATA Task Forces address matters of concern of the global community. There has been no analysis made to my knowledge of the effort wasted due to inadequate attention to data quality during the management of disasters. Suffice to say it must surely be obvious that undue enthusiasm for open science without due attention to research quality or seeking the views of a PI in charge of a given piece of research would be a gross oversight by the authorities managing a crisis or disaster. The need for pre-publication peer review seems an obvious requirement (see *e.g* Rupp *et al.*, 2016 ▸). However, it may be true that in emergencies, the harm of delay might outweigh the harm of error. This has not been analysed to my knowledge or perhaps is even not analysable. There are some useful tools though (https://www.f-uji.net/) from which I quote:

The service is in development, and its assessment depends on several factors.

In the FAIR ecosystem, FAIR assessment must go beyond the object itself. FAIR enabling services and repositories are vital to ensure that research data objects remain FAIR over time. Importantly, machine-readable services (*e.g.* registries) and documents (*e.g.* policies) are required to enable automated tests.

In addition to repository and services requirements, automated testing depends on clear machine assessable criteria. Some aspects (rich, plurality, accurate, relevant) specified in FAIR principles still require human mediation and interpretation.

The tests must focus on generally applicable data/metadata characteristics until domain/community-driven criteria have been agreed (*e.g.* appropriate schemas and required elements for usage/access control *etc*.). For example, for some of the metrics (*i.e.* on Interoperability and Reusability principles), the automated tests we proposed only inspect the ‘surface’ of criteria to be evaluated. Therefore, tests are designed in consideration of generic cross-domain metadata standards such as dublin core, dcat, datacite, schema.org *etc*.

FAIR assessment is performed based on aggregated metadata; this includes metadata embedded in the data (landing) page, metadata retrieved from a PID provider (*e.g.* Datacite content negotiation) and other services (*e.g.* re3data).

## 4. Crystallography as a model of structured openness

I now describe in detail the actions crystallographers and the IUCr, founded in 1948, have taken for transparency and openness in research, and then the work of the IUCr on open educational materials.

### 4.1. Article–data linkage and validation standards

Starting with the first crystal structure publication by Lawrence Bragg (1913 ▸), crystallographers have included Laue diffraction data and monochromatic X-ray diffraction rotating crystal scan data in their publications. This connection of article text with data expanded greatly in the digital era, paving the way for automatic validation checking complemented by specialist referee expertise of a given structural chemistry or structural biology topic. This led to the IUCr’s *checkCIF* reports on the consistency and integrity of crystal structure determinations reported in CIF format and similarly to the PDB validation reports. Linking of articles and data allows reproducibility checks of the authors’ calculations and the checking procedure is a guide to data quality. The crystallography databases ensure the data are FAIR.

**Figure 4:**
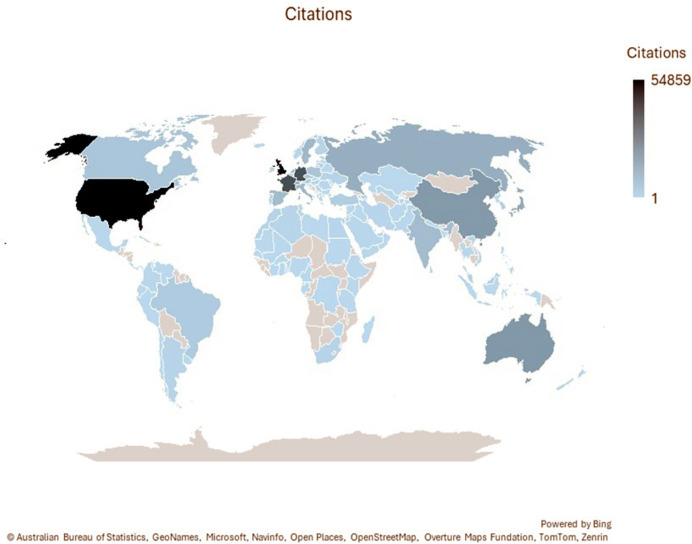
Geographical distribution of published papers in the IUCr Journals, 2016 to 2025. This map and the tabulated data have been provided by Kruna Vukmirovic, Head of Publication Strategy, IUCr.

Crystallography has in fact led the way in transparency of data in publications. Furthermore, IUCr Journals can document a truly global reach across a very large number of countries, within consistent standards, including linking of publications to their underpinning data. Fig. 4 ▸ (see also data in the Supporting Information) shows a map of publications in IUCr Journals by country and the total number of citations.

These maps show a global reach and importantly include articles from industrial companies. *De facto*, IUCr Journals is breaking down Global North and Global South power asymmetries through the large number and wide geographic spread of co-editors; likewise, the IUCr offers wide participation through its General Assembly and provides financial support in the form of conference bursaries and article processing charge waivers. These data also allow the IUCr to see explicitly where training initiatives could have most local impact.

### 4.2. Crystallographic databases and data infrastructure

The Cambridge Structural Database (CSD) (Kennard, 1997 ▸; Groom *et al.*, 2016 ▸) is a pioneer of FAIR data and data of quality since its inception in 1965. It is no longer funded publicly and follows a subscriber-pays model. Recently, individual crystal structures have become free to download but the collection as a whole, which now comprises 1.4 million crystal structures, cannot be downloaded. The Crystallography Open Database (https://www.crystallography.net/cod/), now comprising 0.5 million crystal structures, does allow whole-collection downloads. The PDB is a pioneer of open data: free to depositors and free to access by users. It has received continuous public funding since its formation in 1971. The ICDD (International Centre for Diffraction Data) is a subscriber-accessible high-quality powder diffraction database comprising some 0.5 million entries. These and other databases in the world of crystallography and diffraction are described in detail by Bruno *et al.* (2017 ▸). The evolution of raw data archiving and the growth of its importance in crystallography is described by Helliwell *et al.* (2024 ▸) (see Section 4.3 below). One example is the launch by PDBj (Protein Data Bank Japan) of its raw diffraction data archive, XRDa, which is a significant step forward for reproducibility checks. One such example is described by Helliwell *et al.* (2023 ▸).

### 4.3. Raw data archiving

On the specifics of raw data release for structural biology, the most enthusiastic in the range of crystallography communities to preserve raw data, the community-agreed approach after a decade of deliberation is that there are several steps in the decision to archive. First, a PI judges readiness for submitting for publication, then an editor decides whether to accept it for their journal, and thirdly if a new structure or a new method is a publication’s focus then the raw data should be archived and cited (Helliwell *et al.*, 2019 ▸). IUCr workshops since 2011 have facilitated debate on this topic, with the editors of the IUCr’s structural biology journals describing a policy for authors based on encouragement rather than a mandate.

During the debates in the IUCr workshops, it was realized that there can be a grey area where a PI may wish to share a raw data set with the wider community. Thus *IUCrData* launched a Raw Data Letters category of article (Kroon Batenburg *et al.*, 2022 ▸). Indeed, Raw Data Letters are an excellent vehicle for attracting attention to particularly interesting data sets. Publications of these are now happening across several domains of crystallography.

### 4.4. Personal practice and institutional experiences

Open research practices help ensure that findings are as reproducible as possible. Accordingly, as a laboratory leader, I made all our scientific outputs as available as possible. This involved, as resources grew, open access of our publications, protein structure coordinates and structure factors free to deposit and use via the PDB and, as archiving technology expanded, raw diffraction data via *e.g.* Zenodo. Such an approach made our findings available to all researchers in whatever financial circumstances they found themselves in, useful to Global North and Global South research communities. As a scientific civil servant, I also championed our beamlines at the UK SRS Daresbury being as globally accessible as possible including by telepresence, thus being active in serving the global instrumentation commons (Warren *et al.*, 2008 ▸). In one project theme we developed and extensively validated software (the ‘Daresbury Laue software’) and made the source code openly available (Hao *et al.*, 2021 ▸, and references therein). This action, spanning 30 years, allowed prompt development of synchrotron and neutron Laue macromolecular crystallography at the world’s central facilities. Occasionally our research was of educational and public interest, and we issued press releases and gave open-to-the-public lectures explaining our results. Also, we created the Daresbury Analytical and Research Technical Services (DARTS) for industry to use the SRS beamlines to support their confidential, *i.e.* closed, research in developing and improving their products. A snapshot of the support we provided for the pharmaceutical industry is described by Maclean *et al.* (2006 ▸). Ultimately, as consumers we can choose which company’s product suits us; this I define as a Public Good. I was proud that at SRS, the world’s first dedicated synchrotron X-ray source, we could pioneer this support for industry.

As a career-long contributor in multiple ways to open science, including before it was labelled as such, it concerns me when a facility has an automatic release of raw data after 3 years policy, as this effectively calls into question the judgement of the PI. I may be a rare facility user but I have published the vast majority of the approximately 100 experiments I have performed. In fact, I have had only two failed beamtime runs at a synchrotron, one due to a poor sample and the other due to a too poor calibration to be sure of the reproducibility of the experiments. I imagine that release of those raw data could divert effort unless accompanied by a quality filter, which as a PI I would be happy to give the facility. Indeed, data from failed experiments are not always time wasters (see *e.g*https://oscars-project.eu/projects/fail2fair-recovering-discarded-macromolecular-crystallographic-data). I will return to this issue of unpublished raw data because the central facilities are very concerned about it, and we need to find ways to help them.

Interestingly, in this concept of a spectrum of openness, I note that universities see the need to balance openness and closed as necessary approaches. They charge for teaching and secondly enforce admission standards before admitting students to access knowledge. Universities are often also engaged with IP issues to gain royalties by spin-off companies such as in biotechnology and nanomaterials. They also licence findings to industry as a further ‘funding stream’ opportunity.

Besides my own experiences that I described above, I would welcome other scientists’ descriptions of their open or not-open science experiences.

### 4.5. Open educational resources in crystallography

#### 4.5.1. IUCr Teaching Pamphlets

The IUCr Commission on Teaching in the early 1980s introduced its Teaching Pamphlets on Crystallography Series (Taylor, 1981 ▸). These are classic, open educational resources, free to read and free of any article processing charge to their authors. The IUCr Teaching Pamphlets are fully open to the Global South and Global North. The pamphlets are available at https://www.iucr.org/education/pamphlets. Examples of the topics included are: A non-mathematical introduction to X-ray diffraction, An introduction to the scope, potential and applications of X-ray analysis, Introduction to the calculation of structure factors

The provision of these open educational resources by the IUCr is highly commendable and should be sustained with updating as necessary. Also, with the growing power of quality language translations *e.g.DeepL* these could be made available in the most used languages around the world besides English such as Chinese, Hindi, Spanish, French, Portuguese, Russian and German.

#### 4.5.2. Open textbooks and publishing models

The question also arises whether teaching textbooks can be made diamond open access (https://www.unesco.org/en/diamond-open-access). This is more complicated as costs of production are higher and marketing is likely beneficial. Successful examples of open books are those from University College London Press (https://uclpress.co.uk/), as measured by the very large numbers of downloads of their books.

#### 4.5.3. The IUCr Online Dictionary of Crystallography

The IUCr Online Dictionary of Crystallography (https://dictionary.iucr.org/Main_Page) is free to access online; printed copies are made available for a low-cost payment.

#### 4.5.4. General initiatives of open educational resources

There is a directory of open access books at https://www.doabooks.org/ in which a search on the term ‘crystallography’ yielded at the time of writing 79 hits. The combination of free pdf and a paid for printed copy seems like a good workable model for diamond open access books of the future, in my view.

To come back to a general point to close this section. There is an open educational resources commons available to this day (see https://oercommons.org/). A history of the open educational movement is described by Bliss & Smith (2017 ▸). See also Coursera (https://www.coursera.org/). If certification is wanted, then a fee is charged.

### 4.6. Journal peer review, software transparency, pre-registration and negative results

A relatively easy win for publishers to support a more open science is to make the reviewer report of a publication open. An example of this is my own refereeing report on Langan *et al.* (2018 ▸), see https://static-content.springer.com/esm/art%3A10.1038%2Fs41467-018-06957-w/MediaObjects/41467_2018_6957_MOESM1_ESM.pdf. A pioneer of such open peer review is the journal *eLife*.

The USA National Academy of Sciences, Engineering and Medicine report (2019 ▸) makes clear the value of open software for reproducibility of a study. As I mentioned above, we did this with the Daresbury Laue software from the outset. In 2021 we realized that the software web links had become buried and we deposited the software source code again both at Zenodo and at Cornell University (Hao *et al.*, 2021 ▸, and references therein). Also, there is GitHub, a developer platform that allows developers to create, store, manage and share their software code (https://en.wikipedia.org/wiki/GitHub).

As with other research communities there have been discussions about the value of publishing negative as well as positive crystallization trials or rather creating a database including such. This was achieved for quite a number of years in macromolecular crystallization (Gilliland *et al.*, 1994 ▸, and references therein). A thoughtful and detailed comment on the usefulness of this approach for chemical crystallography was made by Linden *et al.* (2004 ▸) regarding the derivatives of lutein:

Many years ago, a series of well crystallized esters (and a few ethers), most of them of homologous fatty acids, were prepared by Karrer & Ishikawa […]. Lately, we have prepared several of them again and subjected them to various crystallization procedures, such as variation of solvents, application of temperature gradients or diffusion methods, but without success. None of the crystals proved to be good enough for a crystal-structure analysis. Later on, we turned to esters and ethers with more space-demanding properties, e.g. to pivalic acid (=2,2-di­methyl­propanoic acid) or optically active camphanic acid […], but again without success. Finally, the (−)-(1*R*)-menthyl carbonate (1i), prepared from lutein and (−)-(1*R*)-menthyl chloro­formate, a reagent first described by Westley & Halpern […] for the gas-chromatographic separation of racemic amines and alcohols, gave crystals good enough for a complete crystal-structure determination.

So, negative results can be reported in a succinct way rather than a full paper of negative results.

## 5. Conclusions: responsible openness in the next phase

In all my experience serving the IUCr in various roles, as well as my experience as a PI since 1979 and many years involved in central facilities including as a director, I believe that crystallography offers a mature example of structured openness balancing reproducibility, PI responsibility, industrial collaboration, educational outreach and global equity concerns.

Open science cannot be reduced to a simple ‘101’ formulation. As this article has shown, multiple interpretations coexist, and definitions continue to evolve. The Open Science movement’s recent apparent shift at the UN’s Tokyo conference in Otober 2025 from ‘open by default’ to ‘as open as possible as closed as necessary’, now termed Responsible Open Science, reflects a welcome policy maturation. My participation in UNESCO and International Science Council working groups has reinforced my belief that discipline-based traditions of reproducibility, such as those long practised in crystallography, do not always align neatly with broad policy formulations. The wider landscape remains diverse and dynamic, with continuing tensions between openness, intellectual property and responsible governance.

Within this evolving framework, crystallography can still improve. Across all domains of crystallography and related methods, we should pursue systematic article with data peer review, database linkage and validation integrity. At present, this level of rigour is routine mainly in chemical crystallography (including a *checkCIF* report), macromolecular crystallography (including a PDB validation report) and solution small-angle scattering [following the template of Trewhella *et al.* (2023 ▸)]. Extending such practices will require coordinated effort. The other IUCr Commissions could assist IUCr Journals in developing agreed data-quality indicators, while general monitoring of archived raw data sets and software availability could complement detailed peer review where necessary.

More explicit Open Science metrics would also strengthen our position. Possible indicators include: percentage of articles with raw data DOIs; percentage of software made openly available; percentage of negative results reported; participation from Global South countries.

The substantial fraction of unpublished experiments is rightly a concern for central facilities and funders. Wider sharing, as encouraged through initiatives such as PaNOSC, has clear benefits. At the same time, collaboration between facilities and PIs on quality filtering would help distinguish data sets suitable for reuse from those unlikely to yield meaningful results. This approach could reconcile responsible data release with legitimate scientific judgement, while also supporting emerging AI and ML applications that depend on huge data collections. The *IUCrData* Raw Data Letters initiative provides a complementary pathway, allowing PIs to publish raw data sets where they judge them to have value.

Citizen Science represents another area of opportunity. Evidence suggests it is among the most impactful domains of Open Science. Crystallography can build on successful initiatives, such as crystal growth competitions and the Diamond Light Source school-based calcium carbonate project Project M (Murray *et al.*, 2024 ▸) and explore further themes for Open Crystallographic Science. Platforms such as the Open Science Framework (https://osf.io/) may facilitate collaboration and improve the reporting of negative results, which remain underrepresented in the literature.

The broader challenge is how crystallography contributes visibly to the United Nations SDGs. Universities, certainly my own University of Manchester, increasingly map research to specific SDGs; IUCr Commissions and regional or national associations with their Special Interest Groups could adopt similar approaches. Areas such as neglected diseases, where commercial incentives are limited, offer scope for structured, internationally coordinated openness aligned with societal need.

The IUCr already provides substantial global benefit through its open-access journals, Teaching Pamphlets and the Online Dictionary of Crystallography as well as the IUCr Newsletter. These resources should be sustained, updated where necessary and, where feasible, expanded through multilingual translation to enhance accessibility. Publication numbers and citation data associated with IUCr Journals, analysed by country, provide practical tools for planning training initiatives and monitoring their global impact year on year.

Crystallography enters the next phase of Open Science from a position of strength. Its long-standing integration of publication, validation standards and international cooperation provides a tested model of responsible openness and gives strong investment in the global community with its bursaries. By refining metrics, strengthening global inclusivity, enhancing transparency and working constructively with facilities on data stewardship, the crystallographic community can continue not only to respond to evolving policy but to shape it – grounded in proportionality, quality assurance and global engagement.

Overall, UNESCO deserve credit for raising the Global South and North asymmetry and the UN for its SDGs, both in the context of open science. The IUCr has credentials on which it can build and enhance in these domains, thereby contributing even more to the global public good.

## Supplementary Material

Geographical distribution analysis of published papers in IUCr Journals. DOI: 10.1107/S2053273326004146/ae5179sup1.pdf

Duties of a PI. DOI: 10.1107/S2053273326004146/ae5179sup2.pdf

## Figures and Tables

**Figure 2:**
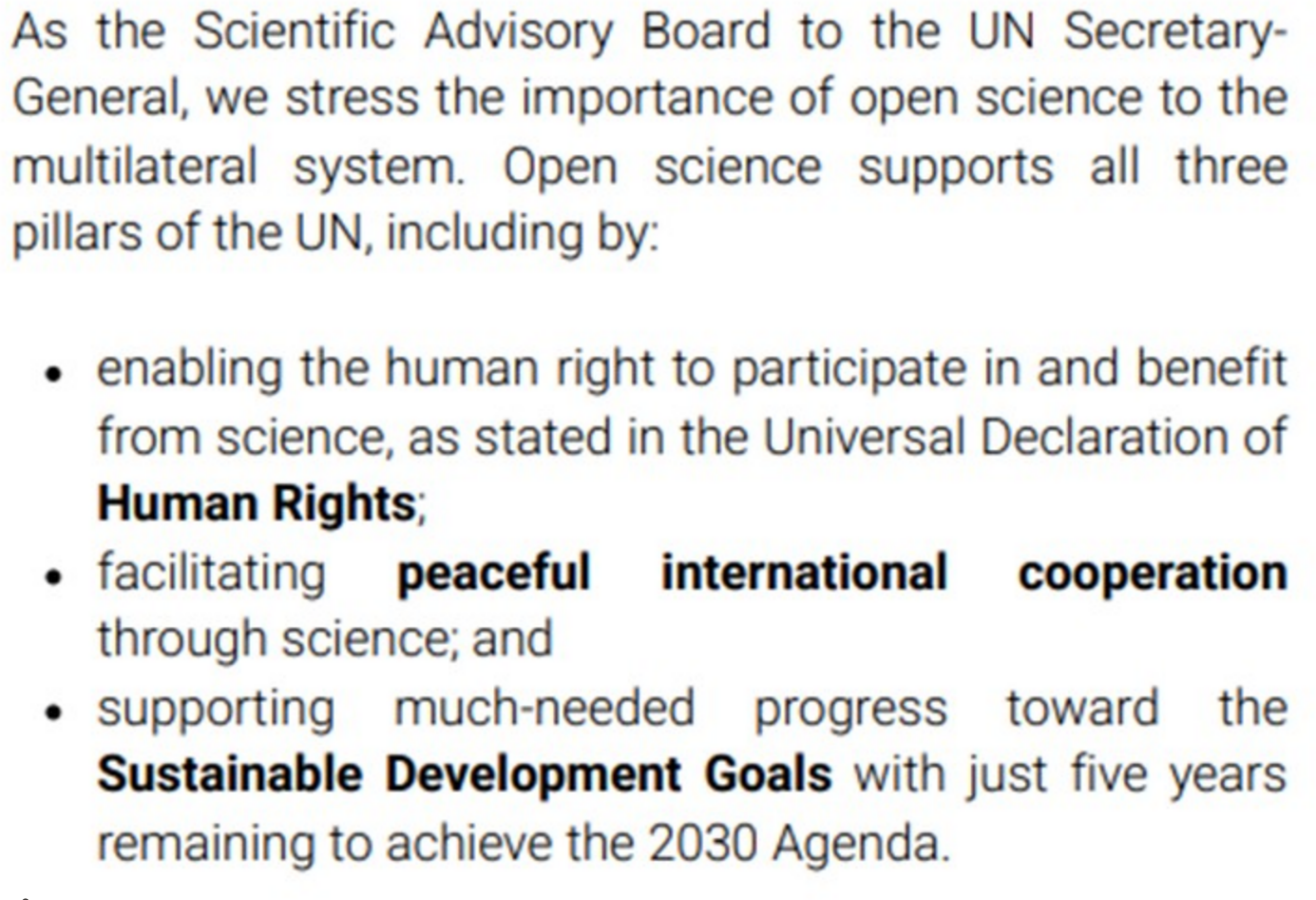
This screenshot from the UN’s Scientific Advisory Board shows Open Science and how it links in a central role to all three pillars of the UN. From these are derived the UN’s Sustainability Development Goals which are very wide-ranging, spanning the Global North’s and Global South’s needs (https://council.science/news/un-scientific-advisory-board-adopts-landmark-statement-on-open-science/).
