# Supplementary material for: Crystallography in Open Science and its open educational resources
Source: Acta Crystallogr A Found Adv. 2026 Jun 16;82(Pt 4):229–41. doi: 10.1107/S2053273326004146 (PMC13325185; doi:10.1107/S2053273326004146)

Acta Cryst A
FOUNDATIONS
ADVANCES

Volume 82 (2026)

Supporting information for article:

Crystallography in Open Science and its Open Educational Resources

John R. Helliwell

S1. Geographical distribution analysis of published papers in the IUCr Journals

Kindly provided by Kruna Vukmirovic, Head of Publishing Strategy, IUCr.

Data Source: Dimensions AI, Digital Science, February 2026.

`Citations' is the total number of citations to the published papers in the analysed period.

`Citations (mean)' is mean citations per published paper in the analysed period.

Table S1 Geographical distribution of published papers in the IUCr Journals, 2016 to 2025 (ordered by the highest citations numbers).

**Country/Region**	**Publications**	**Citations**	**Citations (mean)**
United Kingdom	1685	54859	32.56
United States	3654	51507	14.1
France	1433	32418	22.62
Germany	2273	31929	14.05
Australia	540	15453	28.62
China	1418	15072	10.63
Japan	1071	11197	10.45
Switzerland	538	10048	18.68
Russia	757	9826	12.98
Italy	539	7798	14.47
Sweden	395	7572	19.17
Spain	393	6657	16.94
India	1236	6644	5.38
Netherlands	203	6113	30.11
Denmark	276	5454	19.76
Canada	329	4586	13.94
Poland	500	4311	8.62
Czechia	245	3641	14.86

Brazil	266	3064	11.52
Austria	274	2780	10.15
Turkey	518	2576	4.97
South Korea	336	2364	7.04
Belgium	161	1874	11.64
Singapore	49	1440	29.39
Malaysia	234	1438	6.15
Ukraine	321	1135	3.54
Morocco	406	1097	2.7
Finland	83	1073	12.93
Lithuania	12	1059	88.25
Argentina	77	1051	13.65
Taiwan	113	1002	8.87
South Africa	146	964	6.6
Israel	65	813	12.51
Serbia	26	805	30.96
Luxembourg	20	793	39.65
United Arab Emirates	12	791	65.92
New Zealand	106	772	7.28
Ireland	55	719	13.07
Norway	67	694	10.36
Algeria	138	572	4.14
Egypt	172	560	3.26
Iran	63	551	8.75
Hungary	63	530	8.41
Azerbaijan	151	478	3.17
Saudi Arabia	124	438	3.53
Mexico	130	411	3.16
Croatia	30	408	13.6

Yemen	103	388	3.77
Thailand	84	377	4.49
Romania	82	348	4.24
Portugal	49	332	6.78
Pakistan	47	278	5.91
Tunisia	62	252	4.06
Greece	42	235	5.6
Slovenia	53	231	4.36
Vietnam	70	230	3.29
Slovakia	40	229	5.72
Lebanon	4	216	54
Colombia	86	204	2.37
Uzbekistan	86	181	2.1
Latvia	19	181	9.53
Cameroon	35	163	4.66
Jordan	21	151	7.19
Nepal	82	150	1.83
Senegal	63	131	2.08
Iraq	88	127	1.44
Tajikistan	21	124	5.9
Palestinian Territory	54	123	2.28
Cuba	11	118	10.73
Estonia	6	112	18.67
Uruguay	21	104	4.95
Iceland	5	97	19.4
Chile	22	91	4.14
Bangladesh	40	87	2.17
Malta	10	86	8.6
Ivory Coast	32	80	2.5

Oman	25	80	3.2
Peru	3	71	23.67
Armenia	23	70	3.04
Indonesia	14	58	4.14
Nigeria	15	57	3.8
Bulgaria	13	46	3.54
Democratic Republic of the Congo	7	42	6
Cyprus	8	41	5.12
Burkina Faso	18	40	2.22
Mauritania	17	39	2.29
Tanzania	15	39	2.6
Venezuela	12	39	3.25
Philippines	16	31	1.94
Benin	2	29	14.5
Belarus	13	28	2.15
Gabon	5	23	4.6
Kazakhstan	6	22	3.67
Ethiopia	35	21	0.6
Sri Lanka	13	17	1.31
Andorra	1	15	15
Costa Rica	17	14	0.82
Qatar	5	13	2.6
North Macedonia	3	13	4.33
Kenya	8	10	1.25
Ecuador	5	10	2
Brunei	6	9	1.5
Mali	1	7	7
Moldova	6	6	1
North Korea	3	6	2

Trinidad and Tobago	1	6	6
Kuwait	8	5	0.62
Sudan	2	4	2
Syria	2	4	2
Togo	1	4	4
Zimbabwe	2	2	1
Afghanistan	1	2	2
Djibouti	1	2	2
Montenegro	1	2	2
Libya	3	1	0.33
Ghana	1	1	1
Madagascar	1	1	1
Panama	1	1	1
Albania	1	0	-
Barbados	1	0	-
Bolivia	1	0	-
Kyrgyzstan	1	0	-
Paraguay	1	0	-
Kosovo	1	0	-

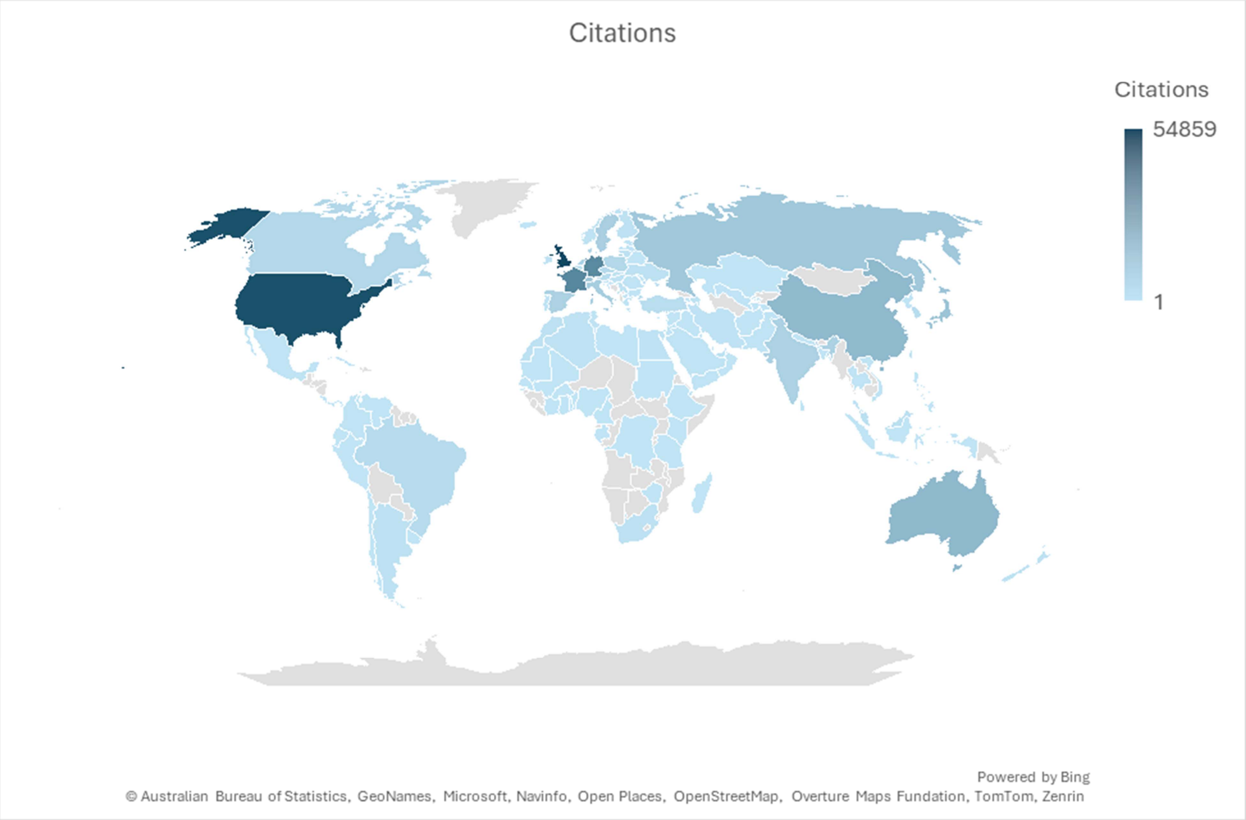

Figure S1 Geographical distribution of published papers in the IUCr Journals, 2016 to 2025. Data Source: Dimensions AI, Digital Science, February 2026.

Table S2 Geographical distribution of published papers in the IUCr Journals, 2016 to 2025 (ordered by the highest citations mean values).

Country/Region	Publications	Citations	Citations (mean)
Lithuania	12	1059	88.25
United Arab Emirates	12	791	65.92
Lebanon	4	216	54
Luxembourg	20	793	39.65
United Kingdom	1685	54859	32.56
Serbia	26	805	30.96
Netherlands	203	6113	30.11
Singapore	49	1440	29.39
Australia	540	15453	28.62
Peru	3	71	23.67
France	1433	32418	22.62
Denmark	276	5454	19.76
Iceland	5	97	19.4
Sweden	395	7572	19.17
Switzerland	538	10048	18.68
Estonia	6	112	18.67
Spain	393	6657	16.94
Andorra	1	15	15
Czechia	245	3641	14.86
Benin	2	29	14.5
Italy	539	7798	14.47
United States	3654	51507	14.1
Germany	2273	31929	14.05
Canada	329	4586	13.94
Argentina	77	1051	13.65

Croatia	30	408	13.6
Ireland	55	719	13.07
Russia	757	9826	12.98
Finland	83	1073	12.93
Israel	65	813	12.51
Belgium	161	1874	11.64
Brazil	266	3064	11.52
Cuba	11	118	10.73
China	1418	15072	10.63
Japan	1071	11197	10.45
Norway	67	694	10.36
Austria	274	2780	10.15
Latvia	19	181	9.53
Taiwan	113	1002	8.87
Iran	63	551	8.75
Poland	500	4311	8.62
Malta	10	86	8.6
Hungary	63	530	8.41
New Zealand	106	772	7.28
Jordan	21	151	7.19
South Korea	336	2364	7.04
Mali	1	7	7
Portugal	49	332	6.78
South Africa	146	964	6.6
Malaysia	234	1438	6.15
Democratic Republic of the Congo	7	42	6
Trinidad and Tobago	1	6	6
Pakistan	47	278	5.91
Tajikistan	21	124	5.9

Slovakia	40	229	5.72
Greece	42	235	5.6
India	1236	6644	5.38
Cyprus	8	41	5.12
Turkey	518	2576	4.97
Uruguay	21	104	4.95
Cameroon	35	163	4.66
Gabon	5	23	4.6
Thailand	84	377	4.49
Slovenia	53	231	4.36
North Macedonia	3	13	4.33
Romania	82	348	4.24
Algeria	138	572	4.14
Chile	22	91	4.14
Indonesia	14	58	4.14
Tunisia	62	252	4.06
Togo	1	4	4
Nigeria	15	57	3.8
Yemen	103	388	3.77
Kazakhstan	6	22	3.67
Ukraine	321	1135	3.54
Bulgaria	13	46	3.54
Saudi Arabia	124	438	3.53
Vietnam	70	230	3.29
Egypt	172	560	3.26
Venezuela	12	39	3.25
Oman	25	80	3.2
Azerbaijan	151	478	3.17
Mexico	130	411	3.16

Armenia	23	70	3.04
Morocco	406	1097	2.7
Tanzania	15	39	2.6
Qatar	5	13	2.6
Ivory Coast	32	80	2.5
Colombia	86	204	2.37
Mauritania	17	39	2.29
Palestinian Territory	54	123	2.28
Burkina Faso	18	40	2.22
Bangladesh	40	87	2.17
Belarus	13	28	2.15
Uzbekistan	86	181	2.1
Senegal	63	131	2.08
Ecuador	5	10	2
North Korea	3	6	2
Sudan	2	4	2
Syria	2	4	2
Afghanistan	1	2	2
Djibouti	1	2	2
Montenegro	1	2	2
Philippines	16	31	1.94
Nepal	82	150	1.83
Brunei	6	9	1.5
Iraq	88	127	1.44
Sri Lanka	13	17	1.31
Kenya	8	10	1.25
Moldova	6	6	1
Zimbabwe	2	2	1
Ghana	1	1	1

Madagascar	1	1	1
Panama	1	1	1
Costa Rica	17	14	0.82
Kuwait	8	5	0.62
Ethiopia	35	21	0.6
Libya	3	1	0.33

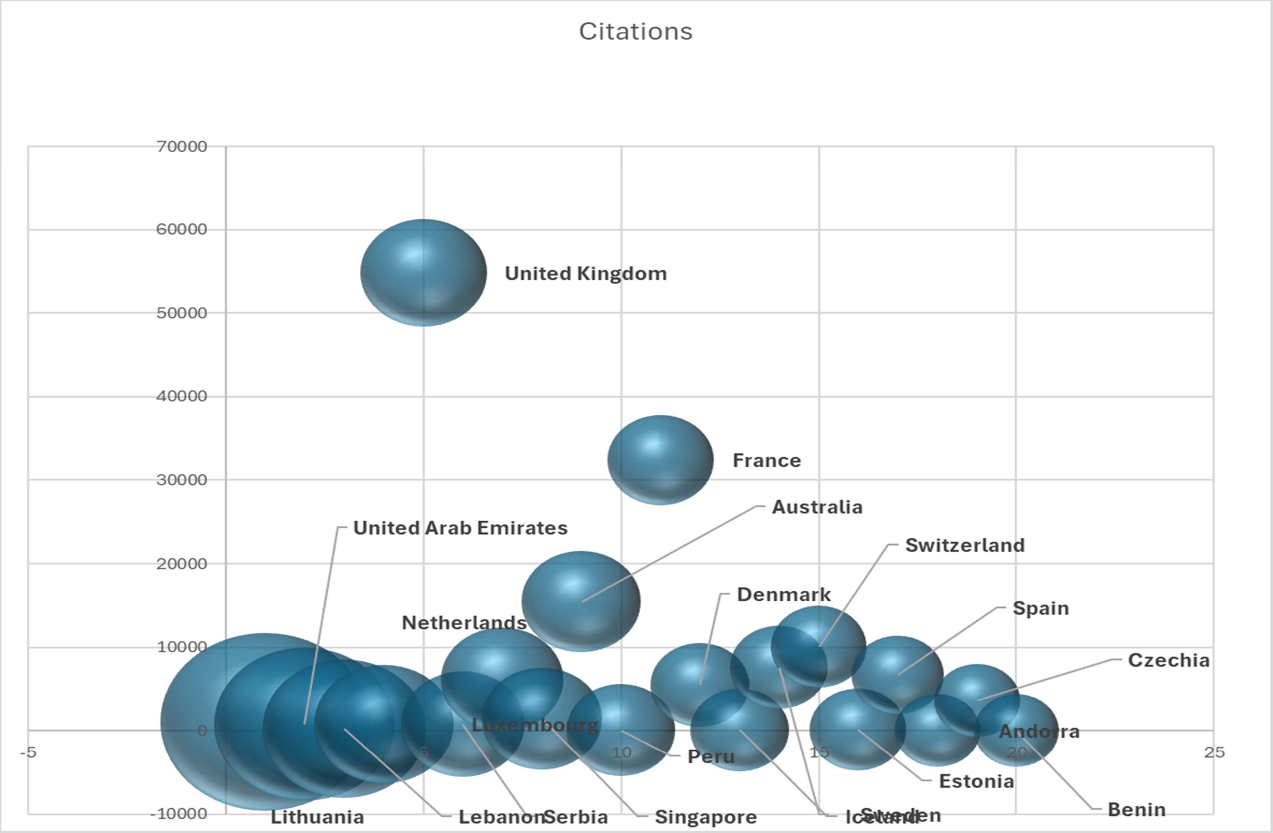

Figure S2 Geographical distribution of published papers in the IUCr Journals, 2016 to 2025 (top 20). Data Source: Dimensions AI, Digital Science, February 2026.

**the size of the bubble corresponds to the citation (mean) in Tables 1 and 2.*

Table S3 Geographical distribution of published Gold Open Access papers in the IUCr Journals, 2016 to 2025 (ordered by the highest citation numbers). Gold open access (OA) means the final published version of the article (or the Version of Record) is freely available online. An article processing charge (APC) is typically required for gold OA publication.
Data Source: Dimensions AI, Digital Science, February 2026.

Country/Region	Publications	Citations	Citations (mean)
United States	1690	9346	5.53
United Kingdom	599	6527	10.9
Germany	978	6377	6.52
India	913	3485	3.82
France	417	3154	7.56
Australia	142	2875	20.25
Japan	409	2569	6.28
Switzerland	191	2529	13.24
China	502	2356	4.69
Netherlands	48	1940	40.42
Sweden	119	1551	13.03
Denmark	67	1543	23.03
Turkey	461	1464	3.18
Malaysia	215	1302	6.06
Italy	152	1293	8.51
Canada	141	1280	9.08
Russia	249	1220	4.9
Morocco	401	1061	2.65
South Korea	198	957	4.83
Poland	111	792	7.14
Spain	91	769	8.45
Singapore	19	566	29.79

Ukraine	256	532	2.08
Czechia	61	522	8.56
Brazil	121	458	3.79
Austria	129	451	3.5
Algeria	112	436	3.89
Egypt	161	428	2.66
Azerbaijan	147	423	2.88
Belgium	62	414	6.68
Yemen	103	388	3.77
Croatia	13	247	19
Mexico	91	238	2.62
South Africa	94	221	2.35
Norway	16	220	13.75
Finland	29	218	7.52
Tunisia	52	216	4.15
Saudi Arabia	103	215	2.09
Hungary	21	206	9.81
Ireland	27	188	6.96
Uzbekistan	82	158	1.93
Nepal	82	150	1.83
Israel	19	144	7.58
New Zealand	44	142	3.23
Senegal	63	131	2.08
Cameroon	28	126	4.5
Tajikistan	21	124	5.9
Vietnam	56	119	2.12
Iraq	86	117	1.36
Palestinian Territory	50	110	2.2
Thailand	54	103	1.91

Taiwan	30	102	3.4
Bangladesh	40	87	2.17
Romania	64	85	1.33
Ivory Coast	31	80	2.58
Jordan	14	78	5.57
Argentina	12	74	6.17
Peru	1	68	68
Portugal	26	61	2.35
Oman	18	60	3.33
United Arab Emirates	4	58	14.5
Slovenia	26	51	1.96
Colombia	39	50	1.28
Pakistan	21	44	2.1
Greece	12	41	3.42
Burkina Faso	18	40	2.22
Mauritania	17	39	2.29
Tanzania	15	39	2.6
Chile	12	35	2.92
Iceland	1	34	34
Indonesia	7	27	3.86
Nigeria	9	25	2.78
Iran	12	24	2
Slovakia	11	24	2.18
Malta	7	24	3.43
Serbia	3	24	8
Gabon	5	23	4.6
Luxembourg	5	23	4.6
Ethiopia	34	21	0.62
Latvia	9	19	2.11

Kazakhstan	4	19	4.75
Cuba	1	17	17
Andorra	1	15	15
Bulgaria	7	12	1.71
Democratic Republic of the Congo	5	11	2.2
Costa Rica	15	10	0.67
Kenya	8	10	1.25
Uruguay	8	8	1
Brunei	5	8	1.6
Moldova	6	6	1
Lithuania	4	6	1.5
Venezuela	3	6	2
Cyprus	1	6	6
Sri Lanka	10	5	0.5
Kuwait	8	5	0.62
Belarus	6	5	0.83
Sudan	2	4	2
Togo	1	4	4
Armenia	2	3	1.5
Estonia	1	3	3
Zimbabwe	2	2	1
Afghanistan	1	2	2
Benin	1	2	2
Djibouti	1	2	2
Libya	2	1	0.5
Qatar	2	1	0.5
Ghana	1	1	1
Madagascar	1	1	1
Panama	1	1	1

Syria	1	1	1
Albania	1	0	-
Barbados	1	0	-
Bolivia	1	0	-
Kyrgyzstan	1	0	-
North Macedonia	1	0	-
Philippines	1	0	-
Paraguay	1	0	-
Kosovo	1	0	-

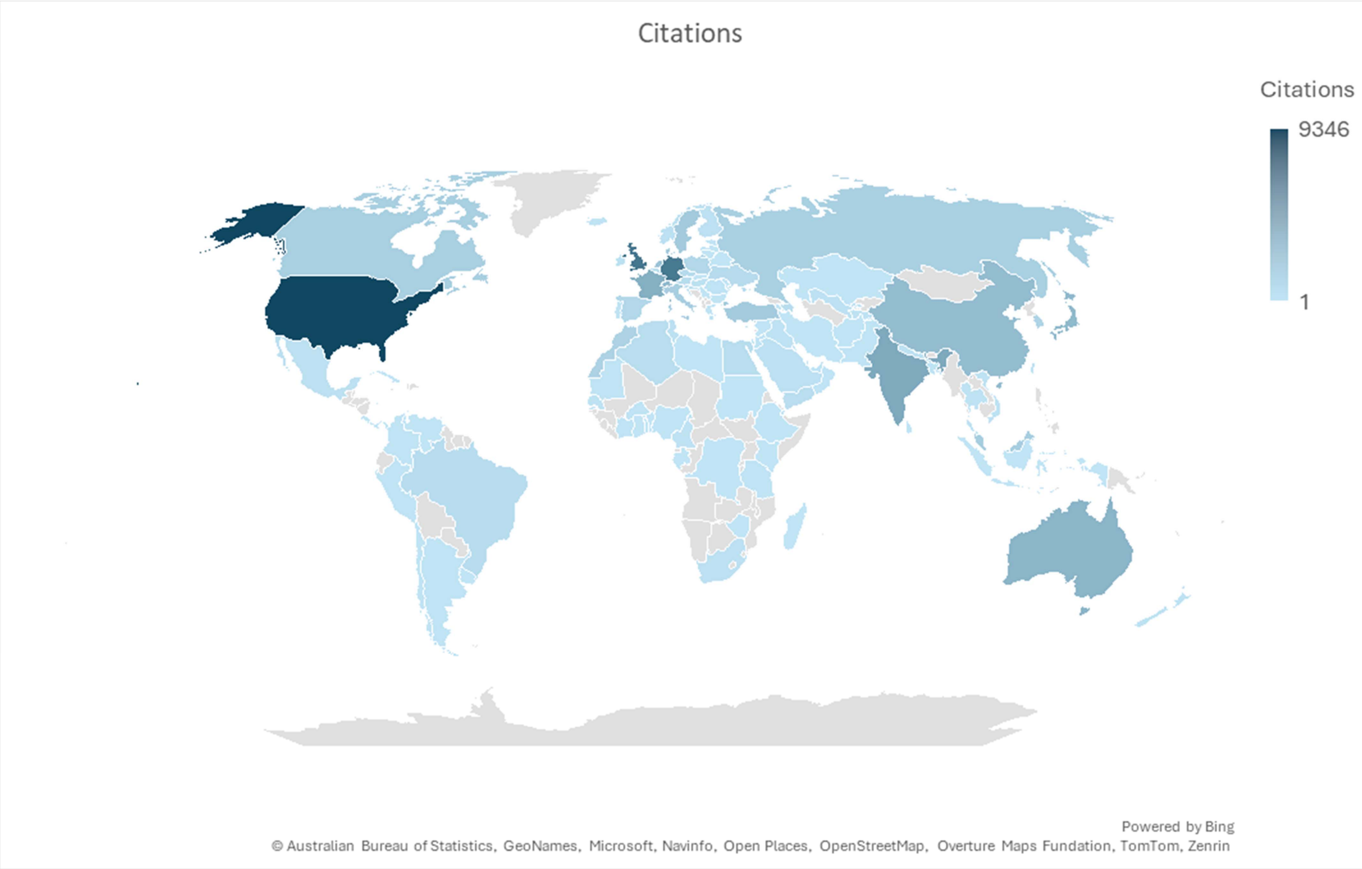

Figure S3 Citation distribution of published Gold Open Access papers in the IUCr Journals, 2016 to 2025 (ordered by the highest citations numbers). Data Source: Dimensions AI, Digital Science, February 2026.

Table S4 Geographical distribution of Gold Open Access published papers in the IUCr Journals, 2016 to 2025 (ordered by the highest citations mean values).

Country/Region	Publications	Citations	Cita-tions (mean)
Peru	1	68	68
Netherlands	48	1940	40.42
Iceland	1	34	34
Singapore	19	566	29.79
Denmark	67	1543	23.03
Australia	142	2875	20.25
Croatia	13	247	19
Cuba	1	17	17
Andorra	1	15	15
United Arab Emirates	4	58	14.5
Norway	16	220	13.75
Switzerland	191	2529	13.24
Sweden	119	1551	13.03
United Kingdom	599	6527	10.9
Hungary	21	206	9.81
Canada	141	1280	9.08
Czechia	61	522	8.56
Italy	152	1293	8.51
Spain	91	769	8.45
Serbia	3	24	8
Israel	19	144	7.58
France	417	3154	7.56
Finland	29	218	7.52

Poland	111	792	7.14
Ireland	27	188	6.96
Belgium	62	414	6.68
Germany	978	6377	6.52
Japan	409	2569	6.28
Argentina	12	74	6.17
Malaysia	215	1302	6.06
Cyprus	1	6	6
Tajikistan	21	124	5.9
Jordan	14	78	5.57
United States	1690	9346	5.53
Russia	249	1220	4.9
South Korea	198	957	4.83
Kazakhstan	4	19	4.75
China	502	2356	4.69
Gabon	5	23	4.6
Luxembourg	5	23	4.6
Cameroon	28	126	4.5
Tunisia	52	216	4.15
Togo	1	4	4
Algeria	112	436	3.89
Indonesia	7	27	3.86
India	913	3485	3.82
Brazil	121	458	3.79
Yemen	103	388	3.77
Austria	129	451	3.5
Malta	7	24	3.43
Greece	12	41	3.42
Taiwan	30	102	3.4

Oman	18	60	3.33
New Zealand	44	142	3.23
Turkey	461	1464	3.18
Estonia	1	3	3
Chile	12	35	2.92
Azerbaijan	147	423	2.88
Nigeria	9	25	2.78
Egypt	161	428	2.66
Morocco	401	1061	2.65
Mexico	91	238	2.62
Tanzania	15	39	2.6
Ivory Coast	31	80	2.58
South Africa	94	221	2.35
Portugal	26	61	2.35
Mauritania	17	39	2.29
Burkina Faso	18	40	2.22
Palestinian Territory	50	110	2.2
Democratic Republic of the Congo	5	11	2.2
Slovakia	11	24	2.18
Bangladesh	40	87	2.17
Vietnam	56	119	2.12
Latvia	9	19	2.11
Pakistan	21	44	2.1
Saudi Arabia	103	215	2.09
Ukraine	256	532	2.08
Senegal	63	131	2.08
Iran	12	24	2
Venezuela	3	6	2
Sudan	2	4	2

Afghanistan	1	2	2
Benin	1	2	2
Djibouti	1	2	2
Slovenia	26	51	1.96
Uzbekistan	82	158	1.93
Thailand	54	103	1.91
Nepal	82	150	1.83
Bulgaria	7	12	1.71
Brunei	5	8	1.6
Lithuania	4	6	1.5
Armenia	2	3	1.5
Iraq	86	117	1.36
Romania	64	85	1.33
Colombia	39	50	1.28
Kenya	8	10	1.25
Uruguay	8	8	1
Moldova	6	6	1
Zimbabwe	2	2	1
Ghana	1	1	1
Madagascar	1	1	1
Panama	1	1	1
Syria	1	1	1
Belarus	6	5	0.83
Costa Rica	15	10	0.67
Ethiopia	34	21	0.62
Kuwait	8	5	0.62
Sri Lanka	10	5	0.5
Libya	2	1	0.5
Qatar	2	1	0.5

Figure S4 Geographical distribution of Gold Open Access published papers in the IUCr Journals, 2016 to 2025 (top 20)

**the size of the bubble corresponds to the citation (mean) in Tables S3 and S4.*

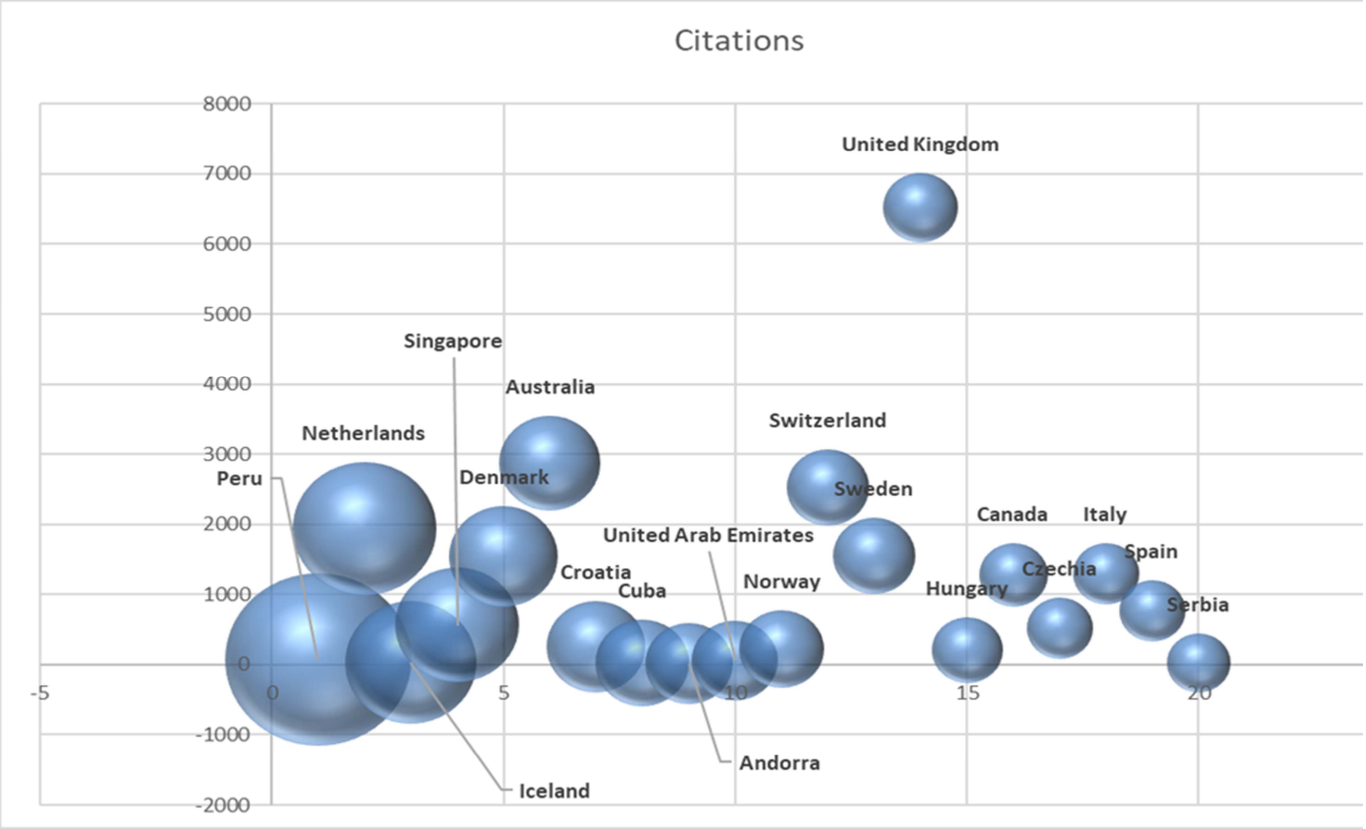

Supplement: Supplementary file 1 [file a-82-00229-sup1.pdf]
