# Supplementary material for: Crystallography in Open Science and its open educational resources
Source: Acta Crystallogr A Found Adv. 2026 Jun 16;82(Pt 4):229–41. doi: 10.1107/S2053273326004146 (PMC13325185; doi:10.1107/S2053273326004146)
Supplement: Supplementary file 2 [file a-82-00229-sup2.pdf]

FOUNDATIONS ADVANCES

Volume 82 (2026)

Supporting information for article:

Crystallography in Open Science and its open educational resources

John R. Helliwell

Duties of the Principal Investigator

Overall categories

**SCIENTIFIC**	**GOVERNANCE**	**LEADERSHIP**	**INNOVATION**
Study design	Ethics approval	Team supervision	Novelty assessment
Data integrity	Regulatory compliance	Mentorship	IP identification
Analysis	Budget oversight	Authorship	Innovation office
Publication	Reporting	Research culture	Patenting strategy

Continuous PI oversight

Continuous PI Oversight, Accountability & Stewardship

(Scientific Integrity • Compliance • Leadership • Translation)

Timeline sense

Timeline Activities

Proposal → Funding → Ethics → Data Collection → Analysis → Innovation Assessment → IP Protection → Publication → Reporting

A PI holds primary responsibility for the scientific integrity, ethical conduct and administrative oversight of the research project.

The PI develops and submits the grant proposal, responds to referees' comments and, in coordination with the host institution, accepts funding and its associated terms and conditions. The PI ensures that all required ethical approvals and regulatory clearances were obtained and maintained, and that the study is conducted in accordance with institutional policies and applicable national legal frameworks.

The PI oversees the design and execution of the study, including the collection, secure storage, processing and interpretation of raw data. The PI is responsible for ensuring data quality, reproducibility and appropriate documentation. Before any public release of data or findings of the research the PI assesses commercial possibilities arising from those either in new methods or applications. If so, the PI informs a university innovation office accordingly with regard to possible patenting or licencing opportunities who would assess novelty and non-obviousness. Decisions regarding data deposition in repositories, preprint submission, authorship and the timing of publication fall under the PI's authority, in accordance with accepted scholarly standards and funder requirements. The PI engages with journal editors and referees during peer review and is the designated Corresponding Author, responding also to any post-publication queries or critiques.

The PI further assumes responsibility for financial stewardship of awarded funds, supervision and mentoring of research personnel, and submission of required scientific and financial reports to the funding agency following detailed consultation with their institution's finance officers.
